# In-Transit Metastasis of Cutaneous Squamous Cell Carcinoma With Lymphovascular Invasion in an Immunocompetent Patient

**DOI:** 10.7759/cureus.21204

**Published:** 2022-01-13

**Authors:** Jacob R Stewart, Ji W Ahn, Jerry D Brewer

**Affiliations:** 1 Dermatology, Mayo Clinic, Rochester, USA

**Keywords:** scc-squamous cell carcinoma, metastasis, in-transit, mohs surgery, cutaneous squamous cell

## Abstract

In-transit metastases of melanoma and non-melanoma skin cancers are metastases located in the skin or subcutaneous tissue between the primary tumor and the nearest nodal basin. Although rare, in-transit cutaneous squamous cell carcinoma (SCC) is an emerging diagnosis in immunocompromised and immunocompetent patients that may have significant implications on treatment and prognosis. Lymphovascular invasion is an uncommon high-risk feature of SCC. Here, we present a case of a 73-year-old non-immunosuppressed man with no previous history of skin cancer, found to have in-transit metastasis with lymphovascular invasion during Mohs surgery for a primary SCC of his right ear. Patients with in-transit SCC should receive further staging imaging and a multimodal approach to treatment, including Mohs micrographic surgery, adjuvant radiation, and possibly sentinel lymph node biopsy and immunotherapy or chemotherapy.

## Introduction

In-transit metastases of melanoma and non-melanoma skin cancers are metastases located in the skin or subcutaneous tissue between the primary tumor and the nearest nodal basin and are hypothesized to occur via lymphatic contamination from the primary tumor [1]. While this form of metastasis has been well described in malignant melanoma, it is seen less often in squamous cell carcinoma [2-4]. Additionally, lymphovascular invasion is an uncommon finding of cutaneous squamous cell carcinoma (SCC) and associated with increased rates of metastatic disease and disease-specific mortality [5]. This case describes an immunocompetent patient with in-transit metastasis and lymphovascular invasion of primary SCC on the right ear.

## Case presentation

**Figure 1:**
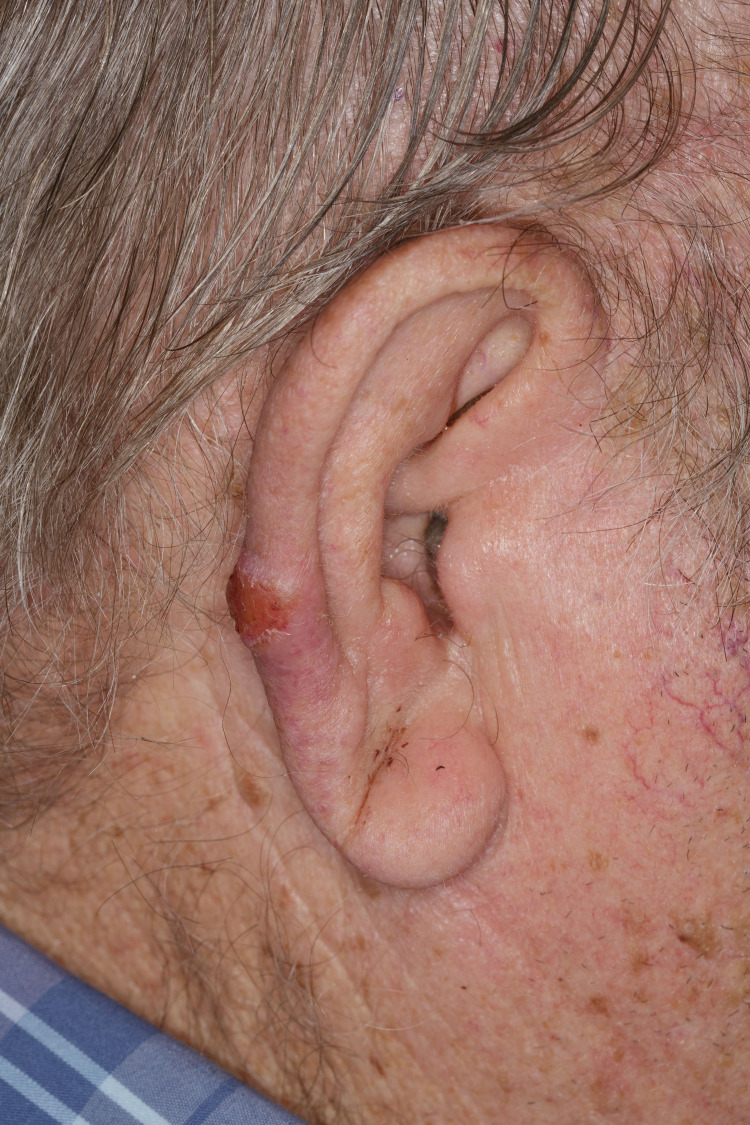
Clinical image shows an erythematous, hyperkeratotic plaque on the right posterior helix.

A 73-year-old non-immunosuppressed man with no previous history of skin cancer presented for Mohs micrographic surgery of a primary SCC on the helix of his right ear. Examination demonstrated an erythematous, hyperkeratotic plaque on the right posterior helix (Figure 1).

**Figure 2:**
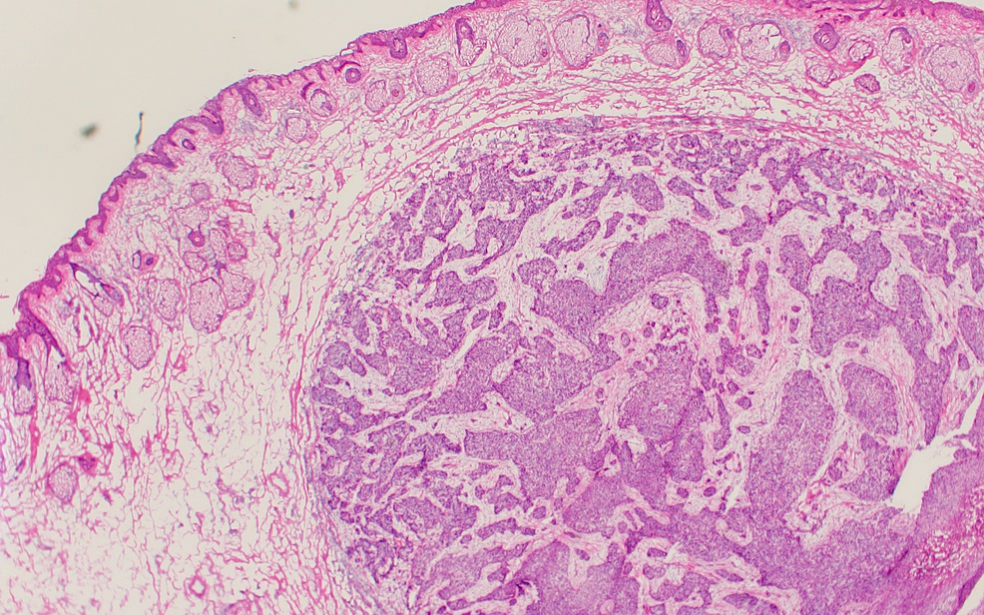
Frozen section histopathology reveals in-transit metastatic nodule of squamous cell carcinoma (hematoxylin & eosin, original magnification ×20).

**Figure 3:**
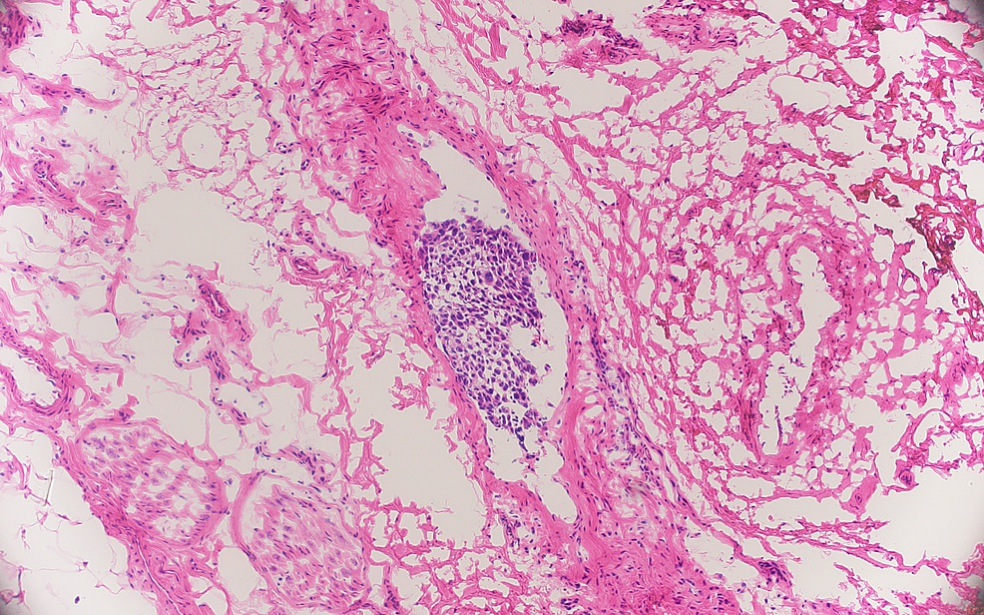
Frozen section histopathology reveals foci of squamous cell carcinoma demonstrating lymphovascular invasion (hematoxylin & eosin, original magnification ×100).

During Mohs micrographic surgery, a subcutaneous tumor was noted midway down the ear toward the neck. Frozen section histopathology of the Mohs micrographic surgery layers revealed a dermal nodule of moderately differentiated SCC, with foci of lymphovascular invasion (Figures 2-3). The in-transit tumor was staged as T2 by the American Joint Committee on Cancer (AJCC) tumor staging criteria and T3 by the Brigham and Women’s Hospital tumor classification system for cutaneous SCC, with moderate to poor differentiation, greater than 2 cm in greatest dimension, high-risk tumor site (ear), perineural invasion (nerve diameter 0.11 mm), and Clark level greater than IV with tumor invasion beyond subcutaneous fat [6,7].

Clear cutaneous margins were obtained with Mohs micrographic surgery, but follow-up positron emission tomography-computed tomography (PET/CT) showed fluorodeoxyglucose uptake in a right intraparotid lymph node, which was confirmed to be positive pathologically for metastatic SCC following superficial parotidectomy and sentinel lymph node biopsy. The patient was referred for adjuvant radiotherapy.

## Discussion

SCCs constitute an increasing proportion of all non-melanoma skin cancers and may lead to serious morbidity and mortality [8,9]. In-transit metastases of SCC are defined as metastatic foci of tumor located between the primary tumor and closest regional lymph node basin and usually present as subcutaneous or dermal nodules [2,4,10]. These metastases usually arise from aggressive primary tumors (usually SCC stage T2 or greater) in immunocompromised patients, such as organ transplant recipients. The primary tumor site is usually on chronic sun-exposed areas or high-risk sites such as the temple, scalp, ear, or lips [2,4]. Differential diagnosis also includes local recurrence, perineural spread, or other metastatic disease.

Given the difficulty distinguishing these diagnoses, diagnostic criteria for in-transit SCC have recently been proposed. The clinical criteria include metastatic lesions that are distinct from previous surgical scars and located between primary tumor and draining lymph nodes. Histological criteria include metastatic tumor being separate from previous surgical scar, histologic similarity to initial tumor, no epidermal origin, and component of tumor outside of perineural locations [4]. Our patient fulfilled all these diagnostic criteria, although his primary tumor was moderately differentiated while the metastatic disease was poorly differentiated in nature. Other cases have also similarly reported worsening tumor differentiation at the site of the in-transit metastasis compared to the primary tumor [3].

Lymphovascular invasion is tumor involving or invading vessels or lymphatics and is an uncommon finding in cutaneous SCC [5]. Furthermore, lymphovascular invasion is an independent predictor of metastatic disease and disease-specific death and thus represents a high-risk histological feature [11-14]. Despite being a predictor of lymph node metastasis, lymphovascular invasion is not included in staging and no studies have yet identified it as having independent prognostic value [5,11].

The relationship between the development of aggressive SCC and immunosuppression, such as following organ transplant, is well established, but the development of in-transit SCC can also be seen in immunocompetent patients [2,10]. The reported prognosis of these patients varies, with disease-specific survival of combined organ transplant recipients and non-organ transplant recipients at three years reported between 27%-56%, and five-year overall survival of 13% [4,15]. One case series, however, reported no deaths at 24 months follow up for non-organ transplant recipients [2]. Further staging with CT or MRI with or without sentinel lymph node biopsy is recommended to evaluate for possible occult disease. Aggressive treatment is usually pursued with a multimodality approach such as Mohs micrographic surgery and adjuvant radiation with or without immunotherapy or chemotherapy. Other management options include decreasing immunosuppression or systemic retinoids [2,4].

## Conclusions

In conclusion, as a recently described phenomenon in immunocompetent patients, in-transit metastases of SCC are hypothesized to represent metastatic disease via lymphatic spread and are associated with an overall poor prognosis. This case is unique due to the histopathological evidence of in-transit metastasis and lymphovascular invasion in an immunocompetent patient. Patients with in-transit SCC should receive further staging imaging and a multimodal approach to treatment, including Mohs micrographic surgery, adjuvant radiation, and possibly sentinel lymph node biopsy and immunotherapy or chemotherapy.
